# Simultaneous Pore Detection and Morphological Features Extraction in Laser Powder Bed Fusion with Image Processing

**DOI:** 10.3390/ma17061373

**Published:** 2024-03-17

**Authors:** Jiaming Li, Xiaoxun Zhang, Fang Ma, Shuxian Wang, Yuanyou Huang

**Affiliations:** 1 School of Materials Science and Engineering, Shanghai University of Engineering Science, Shanghai 201620, China; 2 School of Mechanical and Automotive Engineering, Shanghai University of Engineering Science, Shanghai 201620, China

**Keywords:** pore defect, morphological features extraction, image processing, laser powder bed fusion

## Abstract

Internal pore defects are inevitable during laser powder bed fusion (LPBF), which have a significant impact on the mechanical properties of the parts. Therefore, detecting pores and obtaining their morphology will contribute to the quality of LPBF parts. Currently, supervised models are used for defect image detection, which requires a large amount of LPBF sample data, image labeling, and computing power equipment during the training process, resulting in high detection costs. This study extensively collected LPBF sample data and proposed a method for pore defect classification by obtaining its morphological features while detecting pore defects in optical microscopy (OM) images under various conditions. Compared with other advanced models, the proposed method achieves better detection accuracy on pore defect datasets with limited data. In addition, quickly detecting pore defects in a large number of labeling ground truth images will also contribute to the development of deep learning. In terms of image segmentation, the average accuracy scores of this method in the test images exceed 85%. The research results indicate that the algorithm proposed in this paper is suitable for quickly and accurately identifying pore defects from optical microscopy images.

## 1. Introduction

Additive manufacturing (AM) makes complex parts by building up materials layer by layer, which is opposite to the traditional machining process of removing materials to achieve part manufacturing. LPBF, as a commonly used method in the AM process for manufacturing metal parts, uses a laser as a heat source. The laser will melt the metal powder on the powder bed according to the path set in the computer. After the metal in this layer cools and solidifies, the new metal powder is spread on the previous solidification layer for melting and stacking. Repeat the process continuously until the entire molding process is completed [1,2]. However, the complex thermodynamic interactions during the LPBF process make it inevitable that many defects will form. Cracks and pores are two common defects in LPBF parts [3,4]. Three main types of pores are thought to form during processes: lack-of-fusion (LOF), gas pores, and keyholes (KH) [5]. LOF pores develop from inadequate melting between consecutive layers or adjacent melt trajectories with low laser energy input. They are irregular in shape and larger in size and appear at the boundary of the molten pool [6]. Gas pores are caused by gas being trapped. In the two-dimensional defect representation, they appear spherical and small in size, but numerous and randomly distributed within the molten pool. Under the action of higher laser power, the molten pool will produce a deep and narrow cavity, in which bubbles that fail to rise and escape will eventually be trapped in the lower part of the molten pool to form KH pores. A KH pore is a nearly spherical two-dimensional defect characterization [7,8]. The presence of these pore defects reduces and impacts the mechanical properties to varying degrees [9,10]. Le et al. considered a gas pore generally smaller and spherical, while a lack-of-fusion pore is much larger and irregularly shaped, so lack-of-fusion pores are more harmful to fatigue performance than gas pores [11]. Liu et al. found through experiments that under the same stress amplitude, the fatigue life of equiaxed defects is longer than that of slender defects [12]. Irregularly shaped defects and elongated LOF are more likely to be sources of stress than spherical defects [13,14]. In summary, fast and accurate identification of classified defects allows optimization of critical defect types, which provides an important tool for quality control of the LPBF process. Therefore, the classification and characterization of defects are highly important and have attracted much attention.

Nowadays, deep learning has become increasingly common and has achieved many successful applications in industry [15,16,17]. Wang et al. [18] proposed a neural network defect detection model based on CenterNet for detecting cracks, un-melted powder, porosity, and collapse. After a series of experiments, the model achieves better detection accuracy on defect datasets compared with other models. Caggiano et al. [19] adopted a bi-stream deep convolutional neural network (DCNN) to characterize layer-wise images of the LPBF process for the identification of defects induced by process non-conformities. Cui et al. [20] presented a convolutional neural network (CNN) approach toward robust LPBF quality inspection, such as good quality, crack, gas porosity, and lack of fusion. Wen et al. [21] employed a simple CNN model, YOLOv4 model, and Detectron2 object detection library to achieve successful defect classification, detection, and segmentation, respectively. The CNN model can classify individual cracks or porosity with almost 100% accuracy, while the YOLOv4 and Detectron2 models can identify over 90% of the cracks and pores in the test images. In research on the use of supervised learning to identify defect detection, cracks are elongated and have narrow defects, and LOF pores are very irregular. Both have obvious morphological characteristics, so they are easier to identify and classify. However, in image defect detection models, most do not distinguish between gas pores and KH pores. Because the projections of both are nearly circular, the only distinction lies in the degree of near circularity and size.

Models using deep learning can accurately identify and classify pore defects in images, but the device arithmetic required to train the model, the necessary sample image data, and the ground truth needed tagged time are non-negligible limitations to its development. Supervised learning requires labeling of the ground truth. Distinguishing these slight variances through the human eye proves challenging; different individuals categorizing and labeling defects within identical images cannot fully guarantee the same results. This will inevitably result in inconsistencies and quality issues of the ground truth, which, in turn, will affect the accuracy and reliability of models. Applying shape indicators to categorize pore defects will greatly simplify the workload, and using these indicators to quantify pore defects and define the classification criteria for each pore defect will help avoid classification ambiguity. Thus, this study reports an attempt to detect pore defects in LPBF parts based on the OpenCV method so as to localize pore defects, quantify their morphology, and classify them. In order to evaluate the detection performance of the proposed method, it was compared with current mainstream deep learning models. The study found that the detection performance of our method was significantly better than that of supervised models in a small number of pore defect datasets. At the same time, the average Mean Pixel Accuracy (MPA) and Mean Intersection over Union (MIoU) of image segmentation were 0.8662 and 0.8973, respectively, which were more accurate and robust for pore defect segmentation in images.

## 2. Pore Defect Dataset

**Figure 1:**
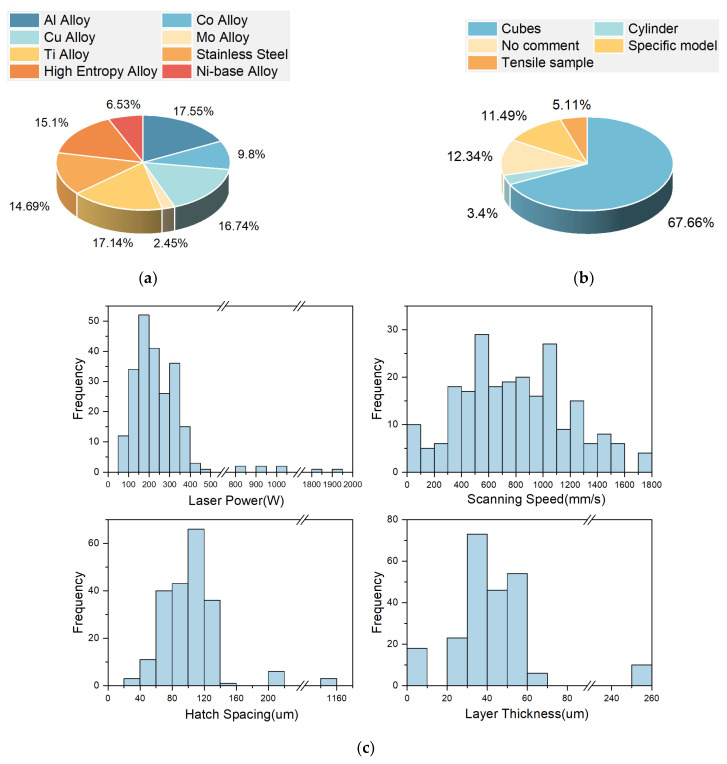
(**a**) The pie chart distribution of materials. (**b**) The pie chart distribution of printed shapes. (**c**) Bar graph distribution of LPBF process parameters.

Investigating the correlation between process parameters and LPBF parts by studying the microstructure is a versatile and effective method [22,23]. The common methods used to collect pore data are XCT for 3D pore data and optical microscopy for 2D pore data. While 3D pore data acquired using XCT provides more information per pore than optical microscopy, in terms of ease of data acquisition and practicality, optical microscopy can provide a faster and more cost-effective way to collect data from multiple samples [4]. Overall, this study used a total of 276 images of pore defects, covering commonly used LPBF materials and process parameter ranges, including stainless steel (304L, 316L), titanium alloys (Ti6Al4V, Ti48Al2Cr2Nb), aluminum alloys (AlSi10Mg, Al7050, Al-Zn-Mg-SC), and more, to ensure that the collected images contain most of the pore morphology [24]. Figure 1 shows the statistical analysis results of some parameters in this pore defect dataset.

## 3. Methods

**Figure 2:**
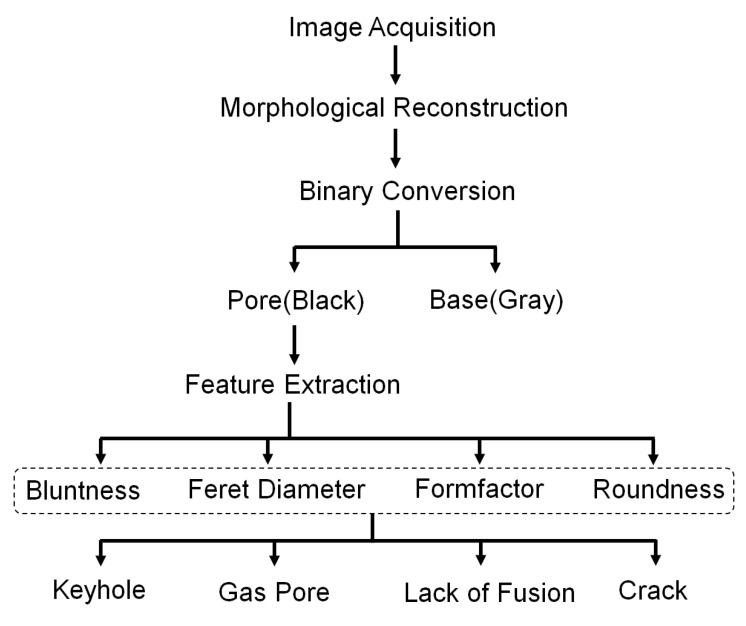
Framework of detection model.

As shown in Figure 2, this study used image processing methods to segment and recognize pore defects in optical microscopy (OM) images. The original OM images were processed in grayscale, and image segmentation of pore defects is achieved through grayscale morphological reconstruction and adaptive threshold segmentation.

**Figure 3:**
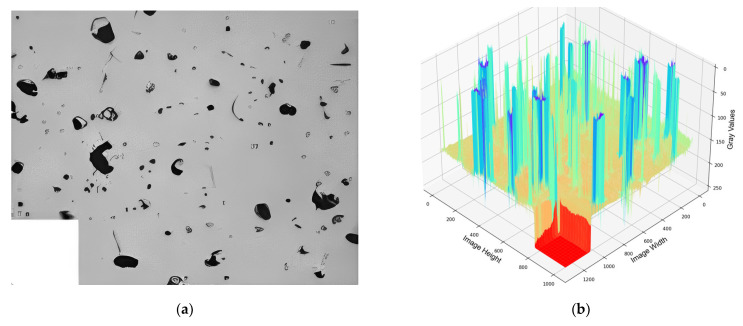
(**a**) The original image. (**b**) The 3D grayscale topographical map of a grayscale image. The blue peak area corresponds to the pore defect, and the red trough area corresponds to the mask.

The information about the pores is obtained from the segmented image to achieve the classification and morphological characterization of the pore defects. The range of the threshold follows the grayscale range from 0 to 255, which gradually presents black to white, respectively. Figure 3 depicts a 3D grayscale topographical map of a grayscale image. It is noticeable that the base of the image has a relatively stable grayscale value. The trough sector correlates to the mask processing region, while the crest region corresponds to the image pores. The presence of differences in gray levels indicates that it is possible to extract data from gray images, consequently setting the base for image segmentation.

### 3.1. Grayscale Morphological Reconstruction

**Figure 4:**
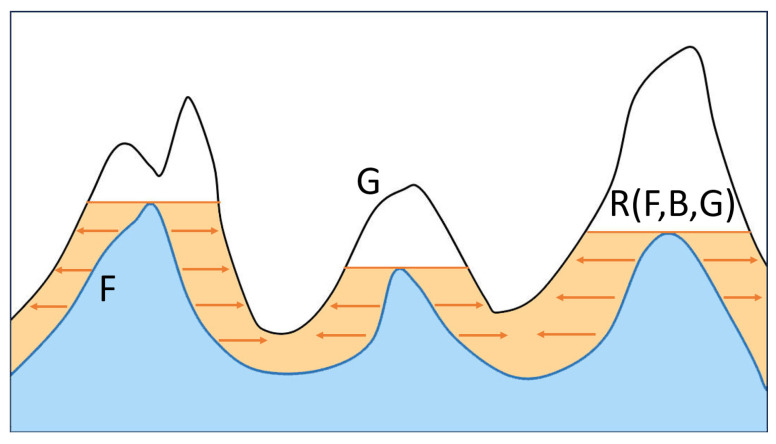
One-dimensional illustration of grayscale morphological reconstruction processing. The seed image intensity profile is defined as F, represented by the blue region. The mask image intensity profile is represented as G, and R(F, B,G) represents the final reconstruction result. The orange area and arrows shows changes in the reconstruction process.

The basic concept behind mathematical morphology involves using different structural elements for measuring and extracting corresponding shapes from digital images, which in turn allows for effective analysis and recognition of images. Grayscale morphological reconstruction, a crucial aspect of mathematical morphology, entails three primary components: seed images containing the starting point for the reconstruction, mask images used to constrain the transformations, and structural elements employed for determining connectivity to create a new image. The grayscale morphological reconstruction process is defined as [25]

(1) $$R\left(F,B,G\right)=\underset{n\to \infty }{\lim}\left[{\left(F⊕B\right)}^{n}\cap G\right],$$

where dilation is a fundamental operation denoted by the symbol ⊕, the iterative dilation of the seed image *F* with the structuring element *B*, constrained by the mask *G*, until stability is reached, as illustrated in Figure 4. The algorithm for grayscale morphological expansion reconstruction involves iteratively applying the dilation operator until the result stabilizes, constrained by the mask image; the reconstruction is reached when the dilated image stops changing, resulting from the reconstruction.

In this study, the grayscale morphological reconstruction is employed to remove pores in the image. It initializes a seed image, sets the values in the last column of the seed image to the maximum value of the original image, expands it by using erosion with a structuring element, and calculates the result by subtracting the filled image from the original image.

### 3.2. Binary Conversation

**Figure 5:**
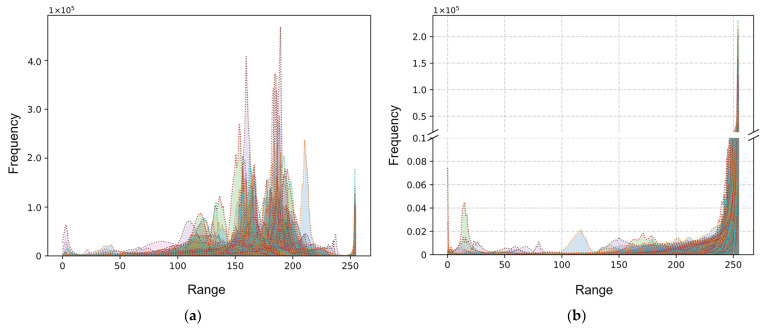
(**a**) The grayscale histograms of all images in the pore defect dataset. Use different colors to differentiate the grayscale histogram of each image. (**b**) Changes in grayscale histogram after grayscale morphological reconstruction.

After the grayscale morphological reconstruction process, the grayscale histogram changes of pore images are shown in Figure 5. It is evident that after processing, the grayscale peaks are concentrated around 250, indicating that the base area has been replaced by white and represents the grayscale peaks of the pores distributed around 0–50. There are obvious double peaks in the grayscale histogram, which indicates a clear separation between foreground and background intensities. Thus, this study selects the Otsu method to find the optimal threshold. The Otsu method, also known as Otsu’s thresholding or maximum variance method, is a classic technique for image segmentation [26]. It aims to search for the threshold that minimizes the within-class variance and maximizes the between-class variance, effectively separating the image into classes with distinct intensity characteristics.

However, Otsu’s method heavily relies on finding distinct peaks in the intensity histogram. For the few grayscale images without obvious double peaks in the dataset, these peaks might not be well-defined, making the optimization process challenging. Therefore, this study uses another technique for determining thresholds. The Yen method calculates the threshold by maximizing a criterion called the Yen criterion, which is defined as a measure of the uniformity of the resulting classes [27]. It is based on the entropy of the pixel intensities and correlation. In low-contrast images, where the intensity values may not exhibit distinct peaks, an entropy-based criterion can still provide a reasonable threshold by considering the overall distribution of intensities, making this method less sensitive to the presence of explicit peaks.

### 3.3. Pore Morphology Indicators

The evaluation and quantification of pores hold great promise in LPBF parts, as the critical effects of their size, shape, and location distribution on mechanical and fatigue properties have been demonstrated [14]. Commonly utilized pore characteristics consist of the diameter and area, which depict the magnitude of the imperfection, and the circularity and aspect ratio, which describe the form [28]. For an accurate classification of pores, information about size and shape is essential.

The first morphology indicator is the Feret diameter (*FD*), which is defined as the distance between two parallel tangents of the particle at an arbitrary angle. The largest *FD* is employed to depict the length of the pore, which is the distance between the two furthest points in the projection direction of the pore.

**Figure 6:**
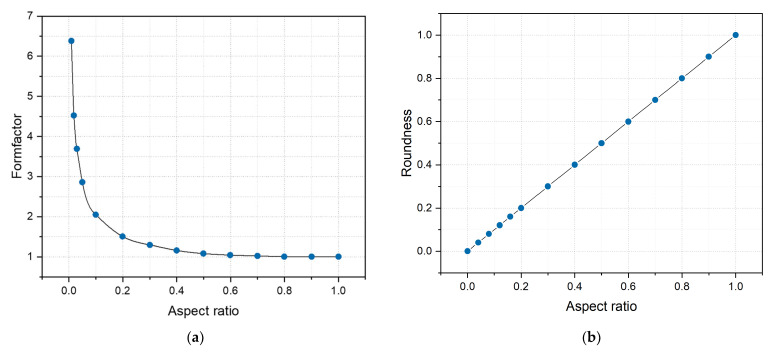
Evaluation of ellipses with different aspect ratios using (**a**) formfactor and (**b**) roundness.

The second morphology indicator is the formfactor (*FF*). It is commonly used as a measure of circularity in various studies [29].

(2) $$FF=\frac{4\pi A}{p^{2}}$$

where *A* is the projected area of the pore, and *p* is the pore’s perimeter. *FF* comprises a projected area and perimeter, and when these two parameters remain constant, the value of *FF* is constant. Nonetheless, the object’s shape may be entirely unlike the original without altering the *FF* value [30]. As depicted in Figure 6a, it is apparent that the *FF* is insensitive to the changes in the shape of the ellipse with an aspect ratio between 0.2 and 1.0, and therefore, they cannot be reliably distinguished from each other [31]. Only using *FF* fails to fully depict the shape of a pore defect.

In order to better characterize the morphology of pore defects, this study introduces new morphology indicators. Roundness (*Rd*) is the extent to which a particle or its projected area approximates a circle in terms of its overall shape [32].

(3) $$Rd=\frac{4A}{\pi d_{max}^{2}}$$

where *A* is the projected area of the pore defect, and $d_{max}$ is the pore’s maximum Feret diameter. With aspect ratios varying from 0 to 1, considering the change curves of the formfactor and roundness, the latter is more capable than the former of distinguishing alterations in elliptical shape resulting from different aspect ratios, as shown in Figure 6 [33].

Irregularly shaped pores have a more significant negative impact on the part compared to nearly spherical defective pores. Therefore, a parameter is necessary to describe the degree of irregularity of the pore profile. Bluntness (*Bt*) is defined as the ratio of the average value of the radius of curvature of the corners of the object outline to the radius of the largest inscribed circle [34].

(4) $$Bt=\left(\frac{1}{n}\overset{n}{\underset{i=1}{\sum }}r_{i}\right)/r_{max}$$

where *n* is the number of corners, $r_{i}$ is the radius of the i-th corner curvature, and $r_{max}$ is the radius of the maximum inscribed circle. From the function formula and Krumbein chart [35,36], it is evident that *Bt* is affected by variations in the edges and corners of the contour. The *Bt* value decreases with an increase in the number of convex parts in the contour.

The pore classification criteria using morphology indicators are shown in Table 1. When *Rd* ≥ 0.7, the aspect ratio exhibits minimal variation concerning the object’s construction direction [37]. The pore defects were first classified as irregular-shaped and circular, and then *FF* and *Rd* were used to differentiate between elongated-shaped cracks. Near-circular gas pores and keyholes were differentiated using *Bt* and *FD* based on the differences between the two, as mentioned above.

## 4. Results and Discussion

### 4.1. Detection of Pore Defects

**Figure 7:**
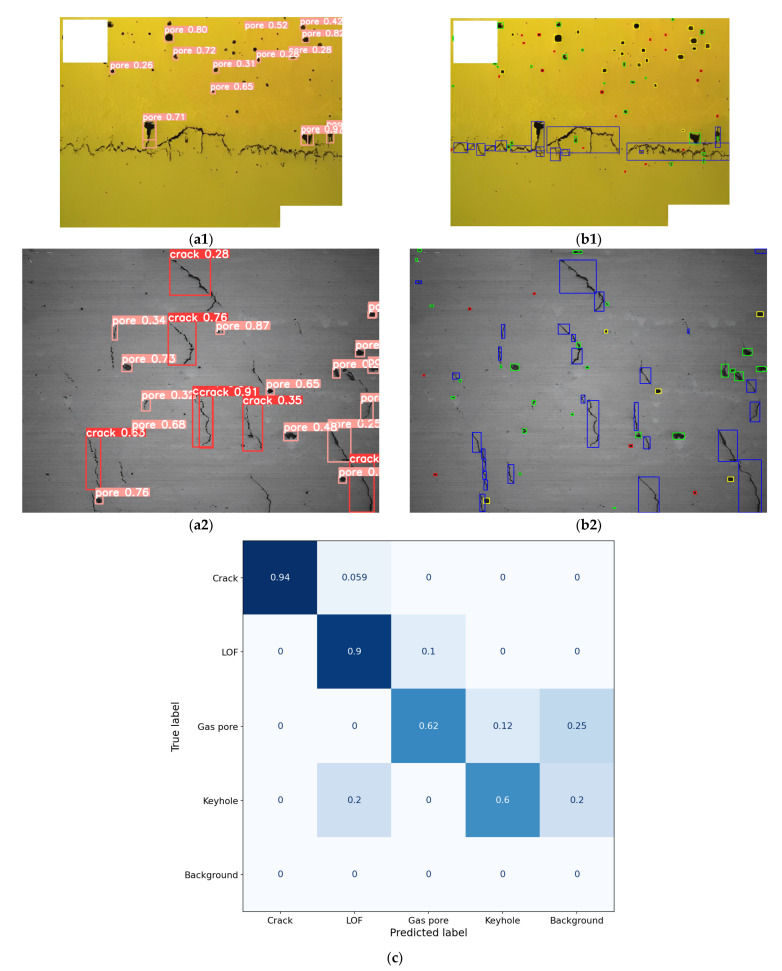
Comparison of the detection result images between the YOLOv8 and our method. (**a**) The detection results of YOLOv8. (**b**) The detection results of our method. (**c**) The confusion matrix of image b2 classification results.

The comparison between the method in this paper and the YOLOv8 detection results is illustrated in Figure 7, which gives the pore defect type and position. Different types of pore defects are indicated by different colors, and locations are indicated by bounding boxes. The method in this paper labels crack as blue, LOF as green, gas as red, and keyhole as yellow. Since labeling ground truth is time-consuming, the training of YOLOv8 is only performed for two categories, namely crack and pores. Pores include LOF, gas pores, and keyholes. There are a total of 60 OM images of LPBF processed samples used for model training, each with multiple cracks and pores. The loss function was optimized by Adam [38] and the initial learning rate was 0.01 for 50 epochs. In order to improve the robustness of the pore defect characterization model, data augmentation is implemented by using random scaling, random flipping, and blurring.

From the results of the comparison, it can be seen that the YOLOv8 model can detect defects with more obvious features in the image, such as the larger pores and cracks in Figure 7a, but the detection effect is poor for most of the smaller pore defects in the images. The obvious transverse cracks in Figure 7(a1) were not identified, possibly due to the limited data on cracks in the dataset. Although data enhancement, such as random scaling and random flipping were used, there is still limited data on transverse cracks, resulting in a limited number of features that the model can learn [20,39]. Compared with other studies, this supervised algorithm has not achieved outstanding detection performance [40,41]. The image data collected in this study have different experimental plans and materials. The defect morphology will be more complex than the image data from the same experiment. The limited training data and a variety of defect morphologies increase the difficulty of accurate recognition. On the other hand, compared with general models, specialized parameter adjustments and structural improvements to the model will achieve better detection results. However, the cost of investment in this attempt cannot be ignored [42,43]. The method in this paper can label most of the pore defects in the image, even the smaller gas pores. Because image processing methods do not require a large number of labeling images for feature learning, appropriate image segmentation methods are used to separate defects from the background of the substrate and then classify them based on defect features. Therefore, the recognition of most defects and smaller defects in the image has been achieved without being limited by labeling data.

With training on a small number of datasets, supervised models cannot obtain satisfactory results even for simple binary classification tasks. Obviously, increasing the training data would be a way to significantly improve the detection performance, but labeling a large amount of image data is bound to be a mechanically repetitive and tedious task for researchers [44]. When labeling the images used to train YOLOv8, the average time to label an image was tested to be about 4 min, while our method takes only about 7 s to label the pore defects. Therefore, in this case, using the method in this paper will save significantly on labor and time costs.

**Figure 8:**
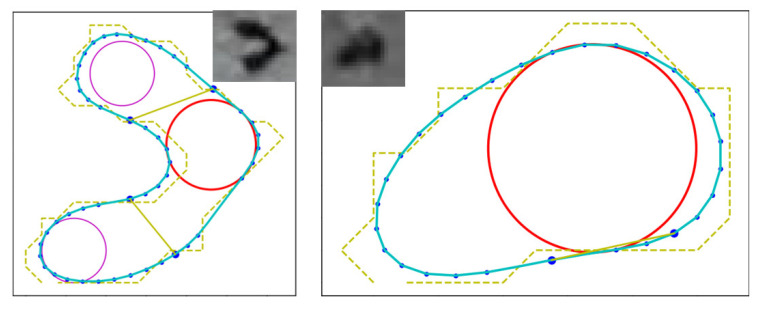
Contour approximation of minor defects. The yellow line represents the defect contour obtained from the image. The blue line represents the fitted contour used for calculating indicators, and the dark blue dots represent the fitted points.

Figure 7c shows the confusion matrix corresponding to the image in Figure 7(b2) for our method; we note the high accuracy of more than 90% for crack and LOF detection. However, the detection of gas pores and keyholes is less accurate. The reason for the limited accuracy is that in order to make most of the defects in the image easier to identify, grayscale morphological reconstruction or binary conversation processing is performed on the image. However, these small defects have too little contrast with the background, and they will be eliminated during these processes, leading to missed detections. On the other hand, their size in the image is too small. When calculating morphology indicators, the number of points used to fit the contour will be limited, as shown in Figure 8. Small sharp corners will be smoothed out due to limited fitting points, which will cause some false detections.

### 4.2. Pore Defect Segmentation 

**Figure 9:**
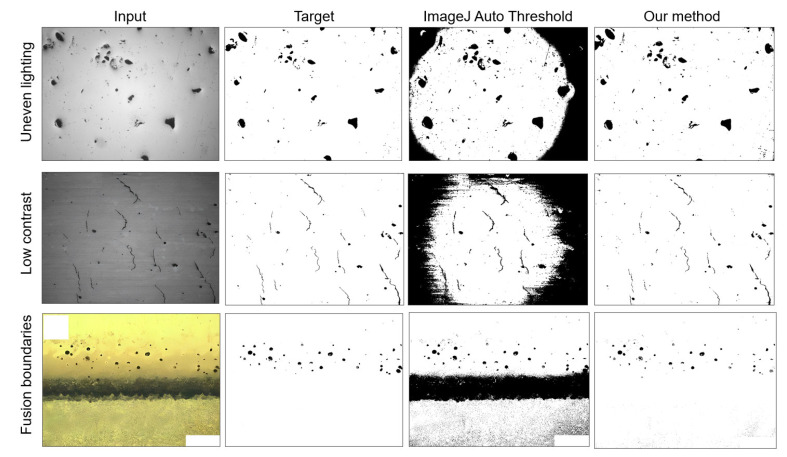
Comparison of segmentation results of test images.

The accuracy of pore defect detection and classification is achieved on the basis of accurate segmentation of the image. The following section describes the results of the comparison of this paper’s method with the default in ImageJ Auto Threshold, focusing on the specific points of the current method in processing complex optical microscopy images. Among the 135 images tested, the default in ImageJ Auto Threshold was used for binarization [45], and 29% of the images had poor processing performance, making it impossible to proceed with the next step of pore defect recognition. Figure 9 selects representative images from low contrast, uneven lighting, and material fusion boundary interference, demonstrating the ability of our method to segment pore defects on complex optical microscopy images. In images with uneven lighting, ImageJ Auto Threshold can clearly segment pore defects in the brighter areas in the middle of the image, but it cannot handle the areas in the darker four corners of the image, resulting in the inability to detect pore defects within them. A similar problem exists in the low-contrast image, where the clearly segmented visible area is smaller and has more noise than the uneven lighting case. For the last case, the interference from the material fusion boundary, using ImageJ Auto Threshold for binarization, the interference of the material fusion boundary allows for the pore defects above the image to be segmented normally, but the fusion boundary is also segmented. Moreover, there are too many noise points below the image, which may be identified as pore defects in subsequent defect recognition, reducing the accuracy of detection. The method proposed in this paper achieves better processing results for the above situations. Grayscale morphological reconstruction changes the grayscale distribution of the image, eliminating most of the interference caused by the matrix background and light shadow. Based on this, a suitable binary conversation is selected for binarization processing, which accurately segments defective parts while avoiding various interferences and improving the robustness of image segmentation.

In order to verify the quantitative results of the pore defect segmentation of the method in this paper, a comparison was made with the binarized target image. The evaluation of image segmentation quality is based on the Mean Pixel Accuracy (*MPA*) and the Mean Intersection over Union (*MIoU*) [46]. *MPA* is the mean of pixel-wise accuracy across all classes. It provides an overall measure of how well the segmentation algorithm is performing, taking into account all classes present in the segmentation task. *MIoU* is the mean of the Intersection over Union (*IoU*) scores across all classes, and the *IoU* for a single class is calculated as the ratio of the intersection of the predicted and ground truth regions to the union of these regions. *MIoU* considers both false positives and false negatives, giving a more nuanced view of how well the segmented regions align with the ground truth.

(5) $$PA=\frac{TP+TN}{TP+TN+FP+FN}$$

(6) $$MPA=\frac{1}{C}\overset{C}{\underset{i=1}{\sum }}PA_{i}$$

(7) $$IoU=\frac{TP}{TP+FP+FN}$$

(8) $$MIoU=\frac{1}{C}\overset{C}{\underset{i=1}{\sum }}IoU_{i}$$

where *FN* is the number of false negative pixels, *FP* is the number of false positive pixels, *TP* is the number of true positive pixels, and *TN* is the number of true negative pixels by the model. *C* is the number of classes, $PA_{i}$ is the Pixel Accuracy for class *i*, and $IoU_{i}$ is the *IoU* for class *i*.

From the results in Table 2, it can be seen that the average *MIoU* of our method is 0.8662, and the average *MPA* reaches 0.8973. This means that even for complex optical images, this method can achieve good image segmentation results.

## 5. Conclusions

This study presents a pore defect detection method based on image processing algorithms, which applies grayscale morphological reconstruction and adaptive binary conversion to optical microscope images, achieving better detection results. By comparing with other advanced models and methods, the following conclusions can be drawn:

1. Compared with the supervised learning network YOLOv8, with a small training dataset, the average detection accuracy of this method is 76.5%, and the detection accuracy for more obvious cracks and LOF defects is over 90%, and it can accurately and comprehensively identify defects in images.
2. The pore defect detection method proposed in this paper will contribute to image data labeling in supervised algorithms, reduce labor costs, and minimize artificial errors to the greatest extent possible. At the same time, the introduction of indicators describing particle morphology quantifies and classifies the characteristics of pore defects, avoiding subjective inaccuracies in pore defect labeling, leading to inconsistent labeling data and quality issues.
3. Compared with commonly used image segmentation algorithms, this method has an average *MIoU* and *MPA* score above 0.85 in complex optical microscopy images. It has stronger robustness and higher segmentation accuracy and can quickly extract the morphological feature information of pore defects.

However, this study still has some limitations. For OM images with more complex lighting conditions, the effectiveness of this method in identifying pore defects is difficult to guarantee; using fixed pore morphological parameters for defect classification can cope with most defect classifications. In the future, with the increase of image data, in order to adapt to changes and improve recognition accuracy, machine learning can be used to adjust the classification standards in a timely manner. In future work, the method in this study may be considered to obtain relevant data information on pore defects and associate it with LPBF printing parameters, providing appropriate references for optimizing manufacturing processes.

## Figures and Tables

**Table 1:** Pore classification criteria using morphology indicators.

Procedure	Pore Defect Class	Formfactor	Roundness	Bluntness	Feret Diameter
First step: Irregular-shaped	Crack	*FF* ≤ 0.3	*Rd* ≤ 0.3	/	/
	LOF	0.3 < *FF* < 0.7	/	/	/
Second step: Circular-shaped	Gas pore	*FF* ≥ 0.7 *Rd* ≥ 0.7		*Bt* ≥ 0.65	*FD* ≤ *FD* average value
	Keyhole			*Bt* < 0.65	*FD* ≥ *FD* average value

**Table 2:** *MPA* and *MIoU* scores using the method proposed in this article on test images.

Test Images	*MPA*	*MIoU*
Uneven lighting	0.8412	0.8366
Low contrast	0.9306	0.8969
Fusion boundaries	0.9202	0.8653

## Data Availability

Data are contained within the article.
